# Observations on Puerperal Fever with Diarrhœa

**Published:** 1814-12

**Authors:** Walter Channing


*For the Medical and Physical Journal.*

### *Observations on Puerperal Fever with Diarrhœa;* by WALTER CHANNING, M.D.

THE name puerperal fever has been long in use to designate a disease, incident to the puerperal state. It is applied equally by all authors, whether they consider the disease as merely inflammatory, or more strictly febrile. It is a disease, let its nature be what it may, (for it is not the design of the author of these observations to determine that,) marked by very striking symptoms, and by a no less remarkable fatality.

In private practice it is of comparatively rare occurrence, and has been found when immediately attended to on its first appearance, to have yielded, in most instances, to such remedies as the symptoms seemed to demand. In hospitals, on the contrary, under every variety of treatment, the fatality which has attended it has scarcely been paralleled by any other disease. It is not my design to give a history of the disease, its symptoms, &c. for these are all detailed in books on practice. My chief object is to relate two cases which have come under my notice, and one of them under my particular care, with such remarks as their peculiar symptoms have suggested.

CASE 1st.—Mrs. M., about forty years of age, the mother of ten children, suffered rather more during her illness with the eleventh, in December, 1812, than with either of the preceding. For some time previous to her confinement, she had been troubled with a cough; not very severe, however, and from which her general health did not seem to be sensibly affected. She was as well as usual the first day after her illness, and on the second, her physician prescribed a cathartic medicine, which her situation required. He called in in the evening, and found her as she thought, under the operation of her medicine. But, the profluvia still being very great, their number being unusually frequent, and the patient very much exposed to cold during each operation, it was judged necessary to exhibit an opiate to moderate or check them. In the morning, however, it was found that they had neither been moderated in frequency or quantity, nor that the patient had slept during the night. The lochia were also found to be diminished. Her pulse was now very frequent and small. The strength very much prostrated and the respiration laborious. There had been chills and heat, succeeded by profuse sweats on the face and upper extremities, circumscribed redness on the cheeks, and the tongue was dry and of a brown colour.

There was but little soreness of the abdomen, and very little tension. Opium in a variety of forms, and tonic stimulant medicines, were exhibited, but with no apparent benefit. I saw Mrs. M. on the morning of the fourth day of her disease, the fifth from her confinement, I carefully counted her pulse, and found them one hundred and sixty beats in the minute, a circumscribed blush on the cheeks, and the face, neck, and upper extremities, covered with that peculiar moisture which is found, more or less, frequently to break out previous to the fatal termination of those diseases, which have been remarkable for excessive evacuations and extreme prostration of the whole system. There was no confusion of mind, and very little soreness of the abdomen, and this was most apparent on pressure. There appeared no one circumstance to warrant a prognosis at all favourable. The stomach appeared irritable from some efforts to vomit, and, from the actual discharge of a porraceous matter, ipecacuanha was given to emesis, and for a time the profluvia were a little diminished; opiates, tinct. of bark and wine combined were prescribed, and a blister was applied over the abdomen. The patient, however, continued to sink, and on the seventh day from the delivery, and on the *fifth* of the disease, she died. Previous to death the tongue was observed to be browner, the moisture mentioned above greater, and a peculiar fœtor, which had been remarked in a slight degree two or three days previous, in the evacuations, was much more apparent. Permission was not obtained to inspect the body, and we have to regret the want of light which dissection might have thrown upon the disease.

Case 2d.—Mrs. A. G., aged about twenty-five, was sick with her fourth child, in March, 1813. She had suffered extremely from a cough, of some months standing, and from abscess abscess of the antrum highmorianum, an operation for which she had submitted to a short time before she fell pregnant. Her cough had been attended with severe pain in the side, for which blisters, bleeding, and antimonial preparations, had been used, with but little effect. This was not one of those affections which at times follow in the train of diseases incident to the pregnant state. It commenced previous to that state, and had been aggravated by exposures, and palliated by remedies, as the whim or necessities of the patient dictated. Two or three months previous to parturition, in a violent paroxysm of coughing, she thought something had been ruptured in the side, and, according to her account, a distinct sound accompanied the supposed rupture. She was bled, vesicated, the cough was quieted, and her general health at the same time improving, she was satisfied her alarms were unfounded. The cough, however, continued during parturition, which last was attended with unusual pain, and was not accomplished by any means as soon as usual. The bowels had been relaxed previous to delivery, for some days, and this continued after delivery. The lochia were more profuse than was common with her, and in the evening of the day of her confinement she nursed her child. She continued well till the beginning of the *fourth* day from her illness. She had, of her own accord, taken a dose of tinct. opii. to quell her cough; on the second day I ordered her, in place of this, the tinct. hyosciami, which I have found to answer better in similar cases. By neither of these means, however, were the usual discharge from the bowels nor the lochia at all checked, when I found her complaining on the fifth day from her confinement, the second of her disease, of pain in the head, heat, flushings, severe pain of the abdomen, and excessive alvine discharges. The pulse was increased to one hundred and forty beats in the minute, and an unusual weakness complained of. The pain of the abdomen was excruciating during the evacuations, and swelling of that part had taken place. I learnt from her nurse that the pain and discharges had appeared the day before; that the first twelve had resembled exactly the usual evacuations of a cathartic medicine; those that followed were compared to the albuminous portion of an egg, and the subsequent ones had exactly resembled those which were now observed; these were almost water, with small masses of mucous matter floating in it. I recommended and urged the necessity of a blister to the abdomen immediately, which was as strongly opposed by the patient, and though she was conscious she must sink under the violence of the diarrhœa, if not moderated, I could only prevail on her to use such internal remedies as might produce that effect. Opiates with astringents were exhibited, but with no effect, and on the third day of the disease all the symptoms were aggravated; the pulse was increased to one hundred and sixty beats in the minute; the face and upper extremities bathed in a cold greasy sweat; the head suffered severe pain; the lochia were checked, and the alvine defluxions so numerous as not to be counted; abdomen swollen and painful to an excruciating degree, and the patient lying on her back unable to move; a consultation was desired, and Dr. Warren, sen. very obligingly attended. I had sent for a large vesicating plaister previous to his arrival, and he agreed with me in the propriety and absolute necessity of its immediate application. It was farther agreed to vomit the patient, and after the operation to continue the use of the opiates, one grain of solid opium, combined with one-half grain of ipecacuanha in pills, one of which was to be given every hour till the pain and alvine defluxions were checked. But little, however, was gained. The patient, though watchful for the two preceding nights, got no refreshing sleep on this; and I exhibited a second emetic on the fourth. Dr. Warren again saw her with me at noon, and it was agreed that some tonic stimulant should be added to the opiate. She passed a very uncomfortable night, but towards morning experienced some diminution of the symptoms. On the *fifth*, a severe itching of the face and breast came on. The pain and discharges were very much lessened, and her pulse, which had fluctuated from the commencement of the disease, between one hundred and forty, and one hundred and sixty, beats in the minute, were found to have come down to ninety-three, were fuller and softer. The pain in the head was lessened, and a pleasant moisture had taken the place of the sweat above mentioned. The vesicated part of the abdomen was the only place where pain was complained of, and the lochia had reappeared. The opium was now gradually diminished in quantity, and soon laid aside altogether, and the patient in a fortnight from this time was as well as puerperal women in general are, who have not suffered the degree of disease just described. Notes of the quantity of various preparations of opium, with the modes of its exhibition, and its combinations with other articles, were not kept. It was, however, very great. It was used combined with chalk julep as an enema. It was also given in form of tincture alone, or combined with tinct. cort. peruv. and cinnamon, and in pills in the solid form. Port wine was craved by the patient, and towards the decline of the disease, and during her convalescence, lescence, she was allowed it in such quantities as were agreeable to her.

There must have been observed a very striking coincidence in the leading symptoms of the above cases. These were in both, excessive alvine defluxions, an extremely morbid secretion of the vessels of the skin, a rapid and small pulse, and great prostration of strength.

It has been distinctly stated, that the pain generally so excruciating, and mentioned, by some authors, as a symptom so strongly pathognononic of genuine puerperal fever, was not present in the case of Mrs. M. It was, however, very remarkable in Mrs. G. Nor was the pain in the head by any means so severe in the former as in the latter. The pulse, however, was in both cases equally remarkable in frequency and smallness. At the close of the disease in the fatal case, it was contracted to a thread ; the natural consequence of the immense evacuations from the system. In Mrs. G. this state of the pulse existing to a degree, and greater at one time than another, according as the discharges were greater or less, indicated her extreme danger. With regard to the treatment adopted in these cases, it may be remarked, that in the last there was the best evidence from the nature and successive changes in the evacuations, that the alimentary canal had been thoroughly cleansed, and the favourable circumstances which occurred were simultaneous, with the alterations produced in the evacuation. The highly morbid action of the skin was very suddenly changed ; and on the fifth day, the critical one of authors who think the disease terminates by crisis, whether fatal or salutary,* a very peculiar action on the skin, in the case of Mrs. G. took place. Whether the severe itching was the consequence of some salutary efforts of nature alone, or a specific action of opium, there may not be data enough to decide. But it is an undeniable fact, that the gradual introduction of a large quantity of that article into the system, has produced this and other effects on the skin, and these, in some cases, at the moment when its good effects were becoming apparent.

To have been influenced by the distracting mass of testimony afforded by books on this puerperal disease, a very different practice might have been adopted, lest the lives of the patients had been endangered, by checking what has been called a salutary effort of nature to resolve abdominal inflammation. But in one case the disease followed literal evacuation by a purgative, and the patient sunk; in the other, no such evacuation was indicated, for the *natural* lax

* Dr. Gordon's Treatise on the Epidemic Puerperal Fever of Aberdeen, p. 74.

state of the bowels continued to the very access of the disease, and became one of its leading symptoms.

It was only moderated by a very liberal use of medicines, whose direct tendency is to diminish all the secretions, and a most rapid return of health supervened on the regulation of these formerly-diseased functions. Mr. White, of Manchester, has given us a case of "puerperal fever with diarrhœa." With this exception, scarce an author has been met with, who has given cases, in which this was a leading and very pressing symptom.

The exciting cause of the disease in Mr. White's patient, (according to his opinion,) did not exist in either of the cases above detailed, viz. the close bad air of the apartment in which the patient was confined.

Authors who have written expressly on this disease, have most of them disagreed as to its nature, its seat, its causes, prognosis, and its cure. Burserius has given a pretty full account of these various opinions, and in the system of midwifery of Mr. Burns, of Glasgow, may be found, in a note to the chapter on the same, a condensed statement of these various and discordant notions. Unquestionably, few diseases have been more fatal than this, under every kind of treatment.

This paper was written solely with a view of stating two cases, whose leading symptoms were of a nature evidently tending to a fatal exhaustion of the living powers, and still bearing a striking resemblance to what has been urged by almost all authors on the subject, as either an effort of nature, and highly dangerous to be interfered with, or, on the artificial production of which, a salutary termination almost exclusively depended. On this account much pains have been taken, many old and new books have been examined, to ascertain how far a diarrhœa and its consequences, (such, let it be particularly attended to, as existed in the preceding cases,) was to be *encouraged*, *moderated*, or *checked*. Riverius, an author quoted by Burserius, was found to have made this remark on the subject, in the last paragraph but one of his chapter "De Morbis acutis puerperarum."—"In generè autem hoc perpetuò observandum; quò longius puerpera distat à die partus, èo tutius medicamentum purgans posse administrari: et contra. Nam experientia docuit, mulieres purgamentorum suppressione laborantes, si post septimum, vel nonum diem alvi fluxu corripiantur, ut plurimum liberari. *Si vero primis diebus, videlicet secundo, tertio, vel quarto diarrhœa acciderit, ul plurimum interire.*" * The notions of

* Riverii opera, Med. Universa, p. 487.

Riverius

Riverius and his cotemporaries, and of many later writers, "de purgamentorum suppressione," viz. that it acts immediately as a cause of the disease under consideration, and of many others peculiar to the puerperal state, have not been established by the best modern writers on the subject; for, though a suppression of the lochia may, and does at times, precede, and frequently attends, the disease in question, still it has been considered a circumstance of accidental occurrence rather than as necessary to the production or continuance of it. For, in many undoubted cases of genuine puerperal fever, not the least derangement has taken place in this uterine function. The practical remark, therefore, respecting the accession of the diarrhœa is that to which particular attention is requested.

Willis, in his chapter on "fevers of women in child-bed," gives it as his opinion that women in this condition are obnoxious to a putrid, or rather malignant, fever, excited by the contagion of a pestilential air. He found that patients were more likely to recover the later the disease occurred from delivery. He did not observe that in his practice it went off by crisis, still the disease was at its height on the fourth day. He dwells particularly on the "vehement and quick pulse," and of "wonderful distensions about the viscera." Sweats were not observed to be favourable, nor *diarrhœa* or the reappearance of the lochia, unless they relieved the symptoms. At page 637 of his translated works, he again observes, that we are not to expect a crisis, "nor does the disease admit the use of a cathartic remedy."— "That if the belly be costive, let it be gently loosened by the violet suppository, or an emollient clyster. *We must beware of a too strong irritation, because it is known that in child-bed the strength is suddenly cast down with a swooning, by copious purging, even as in a malignant fever.*" He concludes by observing, that, although in some very desperate cases, he gained a little by the use of cordial medicines, still when the use of them was for a while remitted, "the diseased fell headlong into death, with a weak pulse, and a looseness forthwith ensuing." *

It is unnecessary to add to the length of this paper by other parallel quotations. The truth indeed is, that, with the

* Dr. Hulme, in his work, has the following remark: "A diarrhœa coming on at the beginning, if followed by a slower pulse, prognosticates safety. But, if after evacuations by stool, whether procured by nature or art, the pulse should not become slower, it is to be reckoned as one of the most dangerous symptoms."—*Hulme on the Puerperal Fever, p.* 31 *and* 32.

 exception exception of Mr. White's case, above alluded to, the occurrence of diarrhœa, such as has been described in the above cases, has been hardly mentioned by authors on puerperal fever. Mr. Burns, indeed, remarks, that "diarrhœa should not be allowed to continue long, and is always to be restrained, unless it *evidently gives* relief, and the fæces be very fœtid." This work, however, is so common, that a bare reference to it is sufficient."

The diversity of opinion which has prevailed with regard to the nature, &c. of the puerperal fever, has been alluded to. The facts which have come under the notice of the author, have not been numerous enough to give a very firm foundation to a new theory, had one been suggested by them. They have afforded additional support to the opinion he has received from various sources, and made it more probable to his own mind, that the disease primarily is purely inflammatory; that this inflammation may and does differ in its nature, and may be seated exclusively in either of the textures of which the intestinal canal is composed; or it may seize the whole of them. It may, for example, seize the peritoneal coat, exciting excruciating pain, constipation, frequent and hard pulse, and, unless removed by such remedies as are indicated, it shall be resolved by large secretions of pus, into the cavity of the abdomen, and this shall destroy the patient, or gangrene may cut her off, before this takes place. It may seize the mucous coat; first excite the canal to the evacuation of its contents, then to an increased secretion of mucous, and thence increasing, waste the patient by diarrhœa, constituted by discharges similar to those which occurred in the case of Mrs. G., unless remedies check it.

Further, this inflammation may attack the omentum or ovaria, constituting the most dangerous varieties of the disease. The constitutional and local treatment proper in the first, must be actively employed in this, and the diseased actions, into which other organs may fall from sympathy, will be occurrences demanding additional regard. There is not room enough in a work of this kind to speculate on the *nature* of this inflammation, and to inquire if this disease be contagious or not, and the questions are not involved in the subject of this paper. It was deemed proper to advert to the varieties of the disease, for, as it has been universally inculcated by all authors, the diarrhœa which affords relief should never be checked; for it is in truth, in most cases, an effort of nature to cure the disease; nay, if there be any evidence of its being the consequence of irritating causes in the intestinal canal, they should be promptly removed by cathartic medicines.

When

When purgatives are indicated, and they most generally are, they should be resorted to as a substitute for what nature is inadequate to effect, in consequence of the violence of the disease. Always, however, bearing in mind that excessive alvine defluxions are among the dangerous symptoms of this disease, and especially when they do not afford relief; and that when they are uselessly, nay, hurtfully excessive, it is our duty, because it is indicated, to check them.—*New-Eng. Journ.*

